# Physiotherapist assisted wrist movement protocol for EEG-based corticokinematic coherence assessment

**DOI:** 10.1038/s41598-025-17330-5

**Published:** 2025-09-26

**Authors:** Fanni Kovács, Adél Ernhaft, Gábor Fazekas, János Horváth

**Affiliations:** 1 Institute of Cognitive Neuroscience and Psychology, HUN-REN Research Centre for Natural Sciences, Budapest, Hungary; 2 Department of Cognitive Science, Faculty of Natural Sciences, Budapest University of Technology and Economics, Budapest, Hungary; 3 Rehabilitation Clinic, Department of Rehabilitation post Stroke, Semmelweis University, Budapest, Hungary; 4 Albert Szent-Györgyi Medical School, Department of Rehabilitation Medicine, University of Szeged, Szeged, Hungary; 5 Institute of Psychology, Károli Gáspár University of the Reformed Church in Hungary, Budapest, Hungary; 6 Institute of Cognitive Neuroscience and Psychology, HUN-REN Research Centre for Natural Sciences, P.O.B. 286, Budapest, 1519 Hungary

**Keywords:** Proprioception, Corticokinematic coherence, Electroencephalography, Assessment, Stroke, Neural encoding, Sensorimotor processing

## Abstract

This study aimed to assess the feasibility of a physiotherapist-assisted wrist-movement protocol to measure corticokinematic coherence (CKC) using electroencephalography (EEG) in healthy adults. The broader goal is to evaluate CKC’s potential as a proprioceptive assessment tool in clinical settings. Thirty-two healthy young adults participated in a hand movement task, in which a physiotherapist periodically moved their hand by relying on a “visual metronome”. We measured CKC by co-recording EEG and hand acceleration data. CKC was observed at the fundamental movement frequency and its first harmonic, mainly at electrodes above the primary sensorimotor area contralateral to the moved hand. The use of a visual metronome helped to maintain the regularity of the movement, but slight between-hand differences in movement regularity were nonetheless present. The results support the feasibility of using a physiotherapist-assisted, wrist-movement-based EEG protocol to assess CKC. This method holds promise for evaluating proprioceptive function in clinical populations, as it enables tailoring the movement to individual needs and real-time adaptation to physiological variability in a natural physiotherapist-patient interaction without requiring a mechanical actuator. The findings lay the groundwork for future applications in stroke rehabilitation and other neurological contexts.

**Supplementary Information:**

The online version contains supplementary material available at 10.1038/s41598-025-17330-5.

## Introduction

 Proprioception – the sense of position, motion and force created by the inertia of one’s own body parts^1,2^ plays a fundamental role in movement control. Although taken for granted, the loss of proprioception leads to difficulties in movement^3,4^. Proprioceptive deficits are especially common after stroke (27–64% of the patients)^5–8^and the level of deficit is correlated with negative outcomes^9^. Clinical tests of proprioception have known challenges with sensitivity, accuracy, reliability, and validity^10,11^thus finding efficient methods to assess proprioception is of key importance^12^ for assessment and rehabilitation. A recently introduced neural measure, which may be suitable for the assessment of proprioception in patients is corticokinematic coherence (CKC^13–15^; for a summary see Bourguignon et al., 2019^16^). CKC reflects synchronization between somatosensory cortical activity and proprioceptive afference produced by continuous, periodic (typically 1–5 Hz) movement or force application^17,18^. In establishing CKC as a neural reflection of proprioceptive processing, previous research (1) has predominantly used magnetoencephalography (MEG) to measure CKC (harnessing MEG’s greater sensitivity to focal cortical activity^19^. Although the viability of the electroencephalography (EEG) -based method has been demonstrated^20,21^and further supported by recent work using EEG in a naturalistic setting^22^more research is needed on the best practices in the application of EEG for the characterization of CKC. (2) Previous research also typically relied on custom-made devices to ensure precise and regular movement, which might not be suitable for certain patient groups (e.g., patients participating in post-stroke rehabilitation). (3) Finally, previous research focused mainly on the movement of the fingers (which have relatively large cortical somatosensory representation^23^. These three experiment design choices plausibly enhance signal-to-noise ratio but may not be viable options in clinical or applied settings. The goal of the present study was to extend the scope of research by exploring beyond these boundaries. We introduced a physiotherapist-assisted wrist-movement-based protocol, which is suitable for patients participating in post-stroke rehabilitation and assess its suitability for the measurement of CKC using EEG in healthy adults.

CKC is observed at the fundamental movement frequency (F0), and its first harmonic (F1 - probably reflecting proprioceptive muscle-spindle signals from agonist/antagonist muscles), mainly in the primary sensorimotor area (SM1) contralateral to the side of the moving body part, but also in other areas, such as the contralateral dorsolateral prefrontal cortex, posterior parietal cortex, and the cerebellar lobule VIII ipsilateral to the movement^13,14,24^.

Although it has also been used to investigate proprioceptive function in individuals with conditions such as Friedreich’s ataxia, the prognostic or diagnostic value of CKC has not been widely explored yet, only a few recent studies have attempted to apply this method in clinical settings: Smeds et al.^20^ demonstrated that CKC can be measured in newborns and potentially used for neurophysiological assessment in neonatal intensive care units. Marty et al.^24^ found a significant decrease in CKC in patients with Friedreich’s ataxia compared to healthy individuals, which remained unchanged in a one-year follow-up^25^.

Notably, EEG-based studies have generally reported lower CKC values than those obtained with MEG. This may be due to MEG’s greater sensitivity to focal cortical activity, or the selectivity of the method to the orientation of the neural sources^19^. Improving methods in extracting CKC from EEG recordings is therefore important for making the measure more accessible and reliable. One promising approach is the use of the surface Laplacian^21^which increases spatial resolution by enhancing the contribution of local cortical activity and reducing the influence of distant, volume-conducted signals on the EEG^26^. To improve signal-to-noise ratio further, the Laplacian is most often calculated by spherical spline interpolation^27,28^ with regularization to smooth the interpolated scalp potentials. The selection of an optimal regularization constant is crucial for accuracy - but this is typically selected for the full processing pipeline, and not for the individual^29^. Whereas a “too aggressive” smoothing parameter may reduce - “smear out” - focal activity, a “too liberal” parameter may not provide a signal-to-noise enhancement. In the present study, to improve signal-to-noise ratio while matching the smoothing parameter to the individual spatial EEG variability, the regularization parameter was chosen individually. As an additional measure to improve spatial resolution, we also interpolated EEG-signals from neighboring electrodes that might have been electrically bridged by excessive application of electrode gel. In such cases the signal in the neighboring electrodes is the same - thus bridging spatially “smears out” the EEG signal. Such bridges are relatively common in EEG recordings. While they may not substantially affect all types of EEG analysis, they can be especially problematic in the present context, where accurate spatial resolution is critical for detecting focal CKC patterns^30^. Previous studies have utilized different devices to produce precise rhythmic proprioceptive afference to measure CKC using pneumatic movement actuators (for fingers^31^; for ankle^32^, but moving the fingers of a child or a newborn could also be done by the experimenters^20,33^. Various movement rates have been employed in CKC research, ranging from 1 Hz to 5 Hz. These different rates have not shown substantial impact on CKC levels or its source location^18^and neither did the amplitude of the movement^34^. CKC can also be obtained by co-recording cortical activity with acceleration, force or electromyogram signals occurring during continuous, periodic movement or voluntary force application^17^.

Although CKC measurements are feasible in various limbs, including the toe or ankle of participants^31,35^most studies have focused on finger movements^17,18,36,37^. Although the relatively large somatosensory cortical representation of the fingers^23,38^ likely contributes to the enhancement of the CKC signal, employing finger movements in clinical research may impose limitations on assessments, as more distal regions of the limbs are often more affected than the proximal ones, particularly in the context of impairments related to stroke^5,39,40^.To accommodate not only normal inter-individual variability, but variability in motor ability as well as in physiological state, for example, different levels of muscle and joint stiffness, spasticity, which often result from stroke^8,41,42^the present study explored the feasibility of a stimulation protocol that involved moving the participant’s hands by a physiotherapist. We investigated whether CKC could be reliably obtained through such a stimulation protocol and produced results commensurate with those reported in the literature. In contrast to a mechanical device moving the body part, the involvement of a physiotherapist would enable individualized movement through one’s own range of motion, in a natural human interaction, ensure ongoing feedback, and allow for prompt adjustments in response to physiological changes (e.g., muscle tension changes, spasms) that may occur during stimulation. For instance, the physiotherapist can pause briefly, or gently modify the movements range or trajectory in response to increased resistance or sudden involuntary contraction. This approach eliminates the need for actuator systems, lowering technical barriers and cost, while supporting clinical applicability by allowing trained therapists to dynamically adapt the stimulation to the participant’s physical condition in real time. Notably, recent work by Mongold et al.^22^ also demonstrated the viability of EEG-based CKC measurement in a naturalistic, actuator-free setting and further linked CKC - especially at the first harmonic of movement frequency - to gross motor performance in healthy adults. Their findings support the behavioral relevance of CKC and complement the present study, which advances clinical translation by addressing the unique needs of stroke rehabilitation contexts through physiotherapist-led stimulation. The moving of the hand by a physiotherapist, however, also creates challenges. In contrast with an artificial movement actuator, the movement imposed by a human agent may well be less regular, which has been shown to reduce CKC^37^. Also, in contrast with moving the finger of a child or a newborn, moving the arm by the wrist of an adult is more difficult. To overcome this problem, in the present study, we introduced a “visual metronome” - a tablet displaying a continuous, periodic animation corresponding to that of a voluntary movement – which was positioned under the moved hand to provide constant visual feedback for the physiotherapist on the regularity of the movement (see *Supplementary Video S1* for a demonstration of the arrangement).

The aim of the present study was to evaluate the feasibility of measuring CKC using EEG in a physiotherapist-assisted, wrist-movement-based protocol in healthy adults. This approach was developed to improve the accessibility of CKC measurements and adapt them for use in clinical populations, particularly in stroke rehabilitation. Unlike prior studies that primarily focused on finger movements, we targeted the wrist, a joint often recovering function earlier than the fingers following stroke, making it a more practical focus for clinical applications. We hypothesized that CKC would be reliably elicited at the fundamental movement frequency (2 Hz) and its first harmonic (4 Hz), primarily over contralateral sensorimotor regions, even in the absence of a mechanical actuator. In addition, we explored whether CKC shows patterns of inter-individual consistency that may reflect underlying trait-like properties.

## Materials and methods

### Participants

Thirty-two healthy young adults (mean [± standard deviation] age 21.9 ± 2.8; range = 19–32; 25 women) participated in the study. Subjects were recruited from a part-time job agency or local universities. They received monetary compensation or course credits in compensation for their participation. They reported having no neurological disorders and normal or corrected to normal vision, and after being informed about the experiment, all participants signed a written informed consent.

Handedness was assessed by the Edinburgh inventory^43^the mean laterality quotient was 77.7 ± 51.3 on the scale from − 100 to 100. 29 participants were right-handed (laterality quotient higher than 40), 1 ambidextrous (laterality quotient − 9.1) and 2 were left-handed (laterality quotient − 100).

### Experimental protocol

The measurements were conducted in a separate room for EEG recording and testing at the National Institute for Medical Rehabilitation (at present Semmelweis University, Rehabilitation Clinic). During the experiment, participants were sitting in an armchair. Two tasks: a Force Exertion and a Hand Movement task was administered. Each task took about ten minutes. In the present paper only results from the Hand Movement task are reported; results from the Force Exertion task will be reported elsewhere. In the Hand Movement Task, a small plastic box with an accelerometer was attached with medical adhesive tape to the back of the participant’s hand, one hand at a time, to record wrist movements. The participant’s hand was occluded from the participant’s view by a curtain. During the task, a physiotherapist (author AE, also partially occluded from view by the curtain) held the participant’s hand in vertical orientation and periodically bent the participant’s wrist at a rate of 2 Hz. The participant’s forearm was fixed to the armrest of the chair by means of an elastic strap, and the physiotherapist held the forearm with one hand. To make sure that the movement period was maintained continuously throughout the task, an electronic tablet (Huawei MediaPad T5 10.1”) positioned under the participants hand playing an animation of a bar moving laterally (i.e., left-to-right, and back at 2 Hz rate) at 30 Hz frame rate. The animation was created on the basis of (50 frame/s) video recordings of voluntary periodic wrist flexions, and then measuring and interpolating hand displacement by cubic spline interpolation over a full cycle of movement. Multiple animations with scaled amplitudes were created, and the animation providing a comfortable movement range for both the participant and the physiotherapist was selected beforehand (total displacement between ~ 2,8 and ~ 10 cm). The participants were instructed to relax with eyes open, and to set their gaze on a fixation puppet placed in front of them to minimize eye-movements during the task, and let the physiotherapist move their hand without them moving a muscle on purpose. Each hand was moved for two 140-second-long periods (blocks - i.e. a total of 280 s per hand), with short rest periods in between as needed. During left hand movements, the physiotherapist sat in front of the participant; during right-hand movements, the physiotherapist sat beside them. The order of the hands was counterbalanced between participants.

## Data acquisition

### EEG

**Fig. 1:**
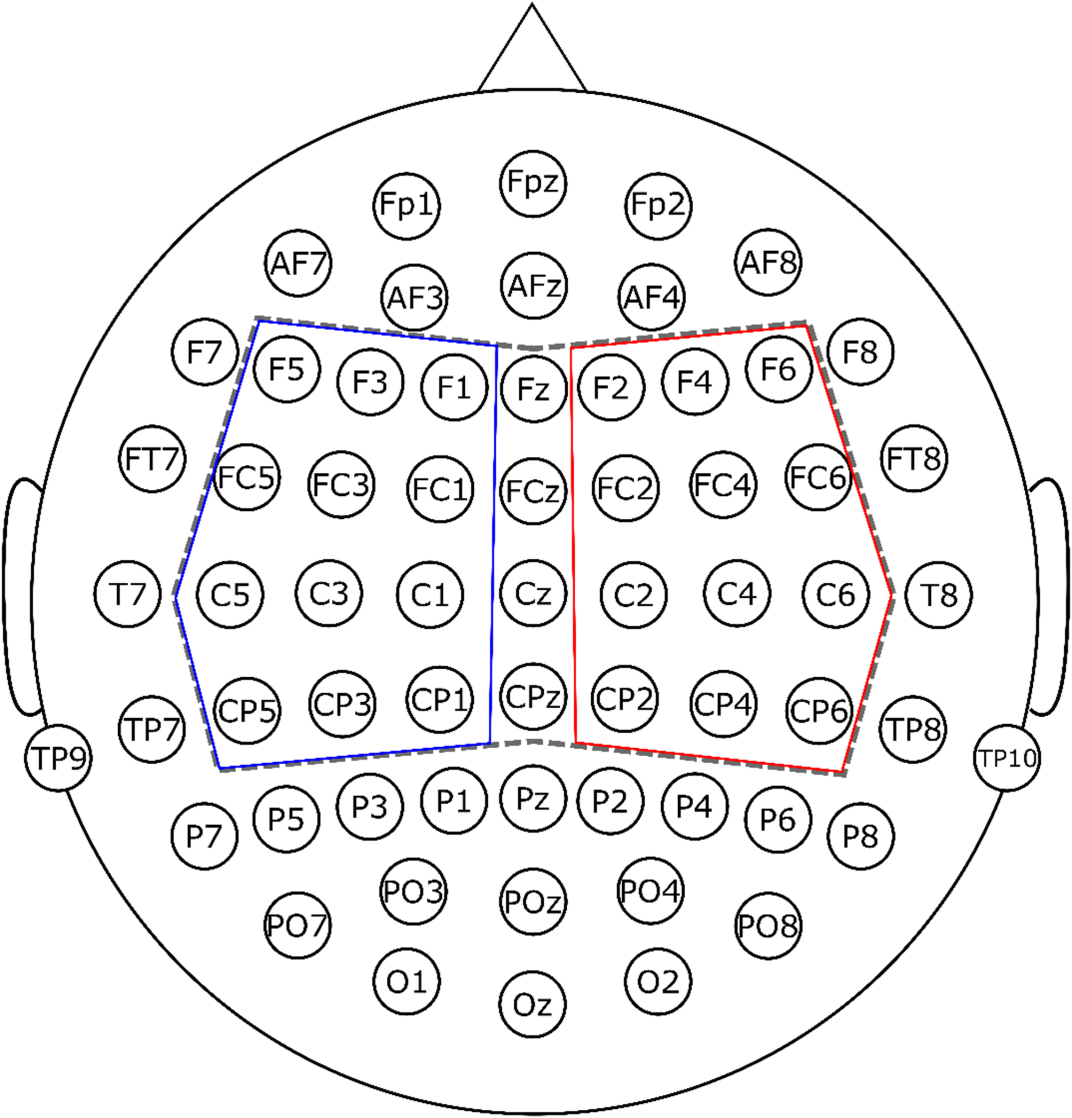
Electrode positions. In the first three experimental sessions 63 electrodes were used - O1 and O2 were not used in later recordings. The grey dashed-line-framed electrodes were used for statistical analyses, blue-framed electrodes were used as left-lateralized, red-framed electrodes were used as right-lateralized in the asymmetry calculation.

EEG was recorded with a Synamp RT (Compumedics Neuroscan, Victoria, Australia) amplifier with a sampling rate of 2000 Hz, and on-line low pass filter at 500 Hz, using a 61 Ag/AgCl sintered ring electrode system (EasyCap, Herrsching, Germany) with electrodes placed according to the 10–10 system^44^ (see Fig. 1) and referenced to the tip of the nose. The electrooculogram was captured by two electrodes placed lateral to the outer canthi of the two eyes in the horizontal dimension, and the Fp1 electrode and another electrode placed under the left eye in the vertical dimension. For the first three participants, 63 electrodes were used, with additional electrodes at the O1 and O2 positions (Fig. 1).

### Kinematics

Acceleration during passive wrist movements were recorded using a LSM6DS33 (STMicroelectronics, Plan-les-Ouates, Switzerland) 3-axis accelerometer (16-bit resolution, with a range set to ± 4 g) encased in a small (33 × 26 × 16 mm) box attached to the back of the moved hand with adhesive medical tape. Acceleration signals were recorded by a separate personal computer with individual samples timestamped. To allow off-line synchronization of the recorded accelerometer and EEG signals, Transistor-Transistor Logic signals were sent to the EEG amplifier. Acceleration signals were sampled at 1094 Hz. Due to a setup error, occasional, random bit flips of the 8th bit for single sampling points occurred, which were detected (as consecutive signal changes exceeding 127 resolution steps in opposite directions) and corrected off-line.

## Data processing and analysis

### Movement signals

Movement analyses were performed using custom Python (3.8.1) scripts relying on the scipy^45^numpy^46^and scikit-learn^47^ software packages. Statistical analysis was performed in R^48^ (version 4.2.1).

To characterize hand movement, the three accelerometer signals were double integrated: in both steps, before the integration step, a high-pass filter (Kaiser-windowed finite impulse response filter, 0.2 Hz cutoff, 0.2 Hz bandwidth, target attenuation of 40 dB, 12209 points) was applied, followed by subtraction of the median signal to compensate for potential drift during the integration (see Starke & Baber, 2017^49^). To capture the main movement axis despite individual variation in the placement of the accelerometer, the resulting displacement signals were submitted to Principal Component Analysis (PCA) and the component with the highest explained variance was selected. After smoothing with a 100-sample-long moving average filter, peaks in this signal were identified by the “peak_find” function of the scikit-learn library, using a minimal distance of 300 samples, and a minimal prominence of 1% of the signal range). Visual inspection confirmed that this procedure successfully captured most signal peaks, with a couple of misses (less than 0.02% of the peaks) due to signal shoulders.

To make sure that potential between-hand asymmetries in lateral movements do not bias comparisons, movement periods were estimated based on maximum as well as minimum peaks. Movement periods and their variability in each block of each participant were characterized by the median and interquartile range of the peak-to-peak intervals, respectively. Both were submitted to two-way repeated measures analyses of variance (ANOVA, implemented in the ez package for R, version 4.4-0^50^) with hand and block number as factors.

To characterize the maintenance of synchrony with the “visual metronome”, the number of consecutive cycles in which all periods were above or below the median period were counted – the duration of such periods would allow characterizing how many cycles of movement it took the physiotherapist to compensate for a drift from the “visual metronome”. Each experimental block was characterized by the median number of cycles before compensation, and these were also submitted to a two-way repeated measures ANOVA with hand and block number as factors. For the ANOVAs, generalized eta squared^51,52^ effect size is reported.

### EEG processing and analysis

Continuous EEG data were processed offline using the MNE-Python toolbox^53^ (version 1.7). To prepare the data for further analysis, certain electrodes were excluded from the raw data. Specifically, due to high levels of noise, the TP9 and TP10 electrodes located behind the ears were removed. Additionally, the O1 and O2 electrodes were excluded from the recordings of the first three experimental sessions to ensure consistency with the later recorded data.

Next, independent components associated with eye blinks were eliminated from the EEG data using the picard method^54^ implemented in MNE-Python. For the independent component analysis, the data was bandpass filtered within the range of 1–180 Hz and decomposed into 61 independent components. Components related to eye movements and blinks (validated through visual inspection of the time-series and topographies) were removed from the original (unfiltered) EEG signal. The number of removed components was 3.031 ± 0.4. The EEG was bandpass filtered between 0.5 and 195.0 Hz. To improve spatial resolution and reduce the impact of electrode bridging, we applied gel-bridge detection and interpolation based on electrical distance metrics^30,53^. A total of 28 out of 32 participants showed at least one bridged electrode pair and up to 10 pairs per subject were interpolated to preserve topographical consistency while limiting overcorrection. Most interpolations occurred over fronto-central and posterior-parietal regions. To optimize the calculation of the surface Laplacian – scalp current source density (CSD) estimation - we used a cross-validation approach to determine the optimal regularization parameter (λ), which balances spatial smoothing and signal fidelity. We tested λ values of 10^−7^, 10^−6^, 10^−5^, 10^−4^ and 10^−3^, with a stiffness value of m = 3.

To reduce computational load, four 50 ms EEG intervals in the middle of the movement recordings were selected. We applied a leave-one-out cross-validation procedure^29,55^ to a subset of 25 centro-parietal and midline electrodes (Fz, F1–F6, FCz, FC1–FC6, Cz, C1–C6, CPz, CP1–CP6, Pz, P1–P6). For each timepoint, one electrode was left out, its signal was estimated via spherical spline interpolation^27,28^and the root mean square error (RMSE) between the estimated and actual signal was computed. This process was repeated for all five λ settings, and the global RMSE across electrodes was used to identify the optimal parameter, which was then used in the CSD estimation, along with 50 Legendre-polynomial terms. The λ values that were used in the final processing varied individually across participants. Specifically, 10^−3^ was used 13 times, 10^−4^ was used 9 times, 10^−5^ was used 5 times, 10^−6^ and 10^−7^ was used 1 time each.

Acceleration signals were synchronized to the EEG, low-pass filtered at 50 Hz and up-sampled to 2000 Hz. For the CKC calculations, the three orthogonal acceleration signals were summarized in two ways: (1) as in previous studies^13,21^ the Euclidian norm was calculated, with accelerometer data corrected by subtracting the median value across time to discount the force of gravity. (2) To capture the main acceleration axis, acceleration signals were submitted to Principal Component Analysis and the component with the highest explained variance was selected. For the calculation of coherence between the CSD-transformed EEG and the acceleration signal, Welch’s method^56^ was applied using SciPy’s signal.coherence function in two ways: (1) the standard approach used a mean-based averaging, whereas in the (2) the modified version, the median across segments was used to reduce the impact of outliers in spectral density estimation.

To characterize CKC signal quality, for the CKC calculation, the CSD-transformed EEG was segmented using 1, 2, 4, and 8 s epochs, each with 80% overlap, leading to 1, 0.5, 0.25, and 0.125 Hz frequency resolutions. For all resolutions, coherence was calculated in four combinations of the two types of acceleration signals (first PCA component or Euclidean norm) and the calculation of the spectral density estimates (mean- or median-based): referred to as (1) *Magnitude-Mean* (2) *PCA-Mean*, (3) *Magnitude-Median* and (4) *PCA-Median* in the following. Spectral estimates were computed using SciPy’s “welch” and “csd” functions, and coherence was calculated using the standard formula:

​ 

$$\:{C}_{xy}\:=\:\frac{{\left|{P}_{xy}\right|}^{2}}{{P}_{xx}{P}_{yy}} ,$$

where ​P_xy_ is the cross-spectral, and P_xx_ and P_yy_ are the power spectral density function of the signals.

For further analysis a subset of electrodes was selected (Fig. 1), including Fz, F1, F2, F3, F4, F5, F6, FCz, FC1, FC2, FC3, FC4, FC5, FC6, Cz, C1, C2, C3, C4, C5, C6, CPz, CP1, CP2, CP3, CP4, CP5, CP6. CKC was characterized at the individual level by the maximum coherence at the fundamental movement frequency of 2 Hz and its first harmonic at 4 Hz across the selected EEG electrodes. The channel showing the highest coherence was determined independently for each participant. Group-level distributions of CKC were visualized using the tidyverse packages in R^57^.

To determine the best method and resolution for further analysis, coherence values were extracted at 2 Hz and 4 Hz - capturing the fundamental and the first harmonic movement frequencies. Coherence at 3 Hz was selected as a baseline measure because this frequency is present across all frequency resolutions tested, and it is close to both the 2 Hz and 4 Hz frequencies of interest. This choice provided a consistent, general reference point for comparing coherence values to quantify how clearly CKC stood out at target frequencies from the baseline across methods and resolutions. Signal-to-noise ratios (SNRs) were calculated by dividing coherence at 2 and 4 Hz by the coherence at 3 Hz and were log-transformed and multiplied by 20 to express results in dB. SNRs were analyzed using a three-way ANOVA with repeated measures factors for Resolution (1 Hz, 0.5 Hz, 0.25 Hz, 0.125 Hz), Feature Extraction Method (PCA vs. Magnitude), and Averaging Strategy (Median vs. Mean). These analyses identified the combinations that consistently produced strong, frequency-specific coherence responses, supporting the selection of an optimal setup for downstream analysis. When the sphericity assumption was violated as indicated by significant Mauchley’s tests, degrees of freedom were Greenhouse-Geisser-corrected.

After identifying the optimal combination of resolution and coherence estimation method, all subsequent statistical analyses were performed exclusively on data derived from this configuration to assess condition-related differences in CKC, its hemispheric distribution, and its relationship to handedness.

To characterize the typically asymmetric lateralization of CKC (as described in the literature), mean coherence across the selected electrodes were individually calculated on the left (F5, F3, F1, FC5, FC3, FC1, C5, C3, C1, CP5, CP3, CP1) and right (F2, F4, F6, FC2, FC4, FC6, C2, C4, C6, CP2, CP4, CP6), at 2 and 4 Hz, contra- and ipsilateral to the moving hand.

Due to the non-normality of the data as indicated by Shapiro-Wilk tests, Wilcoxon signed-rank tests were calculated.

To assess frequency specificity of coherence across different frequencies, coherence values were compared at target frequencies (2 Hz and 4 Hz) with neighboring frequencies (1.5, 2.5 Hz for 2 hz and 3.5, 4.5 Hz for 4 Hz). Paired t-tests, Wilcoxon signed-rank tests, and Shapiro-Wilk tests for normality are applied to determine whether coherence at the target frequency significantly differs from the neighboring frequency.

The relationship between the CKC measured in right- and left-hand conditions at 2 Hz and 4 Hz was characterized by Spearman correlations. Additionally, the same correlations were examined separately for the dominant hand and non-dominant hand conditions. For the one ambidextrous participant, the dominant hand was determined to be the left hand based on self-reported handedness and the laterality quotient (−9.1) indicating a slightly stronger left preference. Furthermore, correlations between handedness and the laterality quotient were examined.

## Results

### Movement analyses

Participants mostly complied with the instructions, with occasional difficulties in relaxing the moved arm reported by the physiotherapist in 9 blocks (three left-hand and six right-hand movement blocks − 8 participants; out the total of 128 blocks of 32 participants).

The ANOVA of the estimated movement periods showed a significant main effect of hand side: *F*(1,31) = 14.245, η^2^_G_ = 0.135, *p* < 0.001 (499.7 and 499.1 ms, respectively for the left and right hands, with a mean difference of 0.60 ± 0.90 ms). Neither the block main effect (*F*(1,31) = 0.089, η^2^_G_ = 0.0007, *p* = 0.767), nor the interaction effect (*F*(1,31) = 1.433, η^2^_G_ = 0.008, *p* = 0.240) was significant.

The ANOVA of the inter-quartile ranges of movement periods showed a significant hand side main effect: *F*(1,31) = 10.076, η^2^_G_ = 0.115, *p* = 0.003 (20.50 and 25.53 ms respectively for the left and right hand, with a mean difference of 5.03 ± 8.97 ms). Neither the block main effect (*F*(1,31) = 0.539, η^2^_G_ = 0.001, *p* = 0.468, nor the interaction effect (*F*(1,31) = 0.244, η^2^_G_ = 0.0004, *p* = 0.624) was significant.

The ANOVA of the median number of cycles before compensation showed no significant effects (hand side main effect: *F*(1,31) = 0.244, η^2^_G_ = 0.002, *p* = 0.625; block main effect: *F*(1,31) = 2.385, η^2^_G_ = 0.026, *p* = 0.133; interaction effect: *F*(1,31) = 3.207, η^2^_G_ = 0.026, *p* = 0.083), with the mean number of cycles before compensation across blocks being 1.187 ± 0.392.

### Coherence analyses

**Fig. 2:**
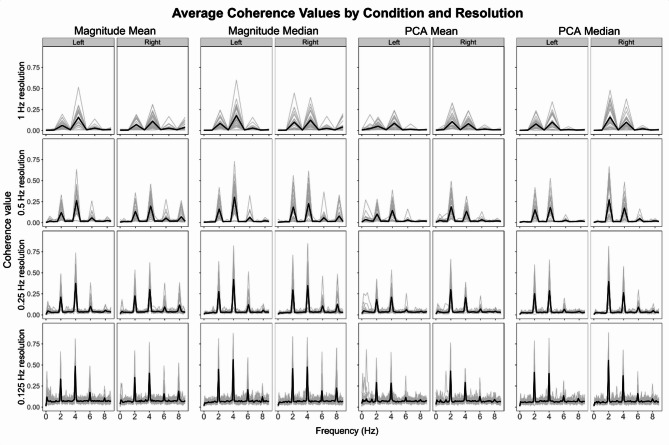
CKC spectra between wrist acceleration and EEG signals averaged across all participants (*n* = 32) in all methods (columns) and resolutions (rows). Coherence peaked at the 2 Hz-movement frequency and its harmonics. Black solid lines indicate the group-mean, and gray lines indicate individual coherences. Coherence consistently peaked at the 2 Hz movement frequency and its first harmonic (4 Hz), even at the finest resolution (0.125 Hz), indicating robust frequency-locked cortical responses across all processing variants.

The individual (thin gray lines) and group-mean (thick black line) CKC spectra across frequencies from 0 to 8 Hz are presented in Fig. 2, separately for each method (columns), resolution (rows), and movement condition (right vs. left hand). For each frequency bin, the highest coherence value observed across all selected EEG electrodes is shown. Across all methods and resolutions, clear CKC peaks emerged at 2 Hz - the fundamental movement frequency - and at its first harmonic (4 Hz), suggesting that the passive wrist movement was temporally precise and rhythmically stable across participants. The stability of these peaks - even at high spectral resolution (0.125 Hz) - supports the viability of our protocol for generating temporally precise proprioceptive stimulation in a clinically adaptable manner.

**Fig. 3:**
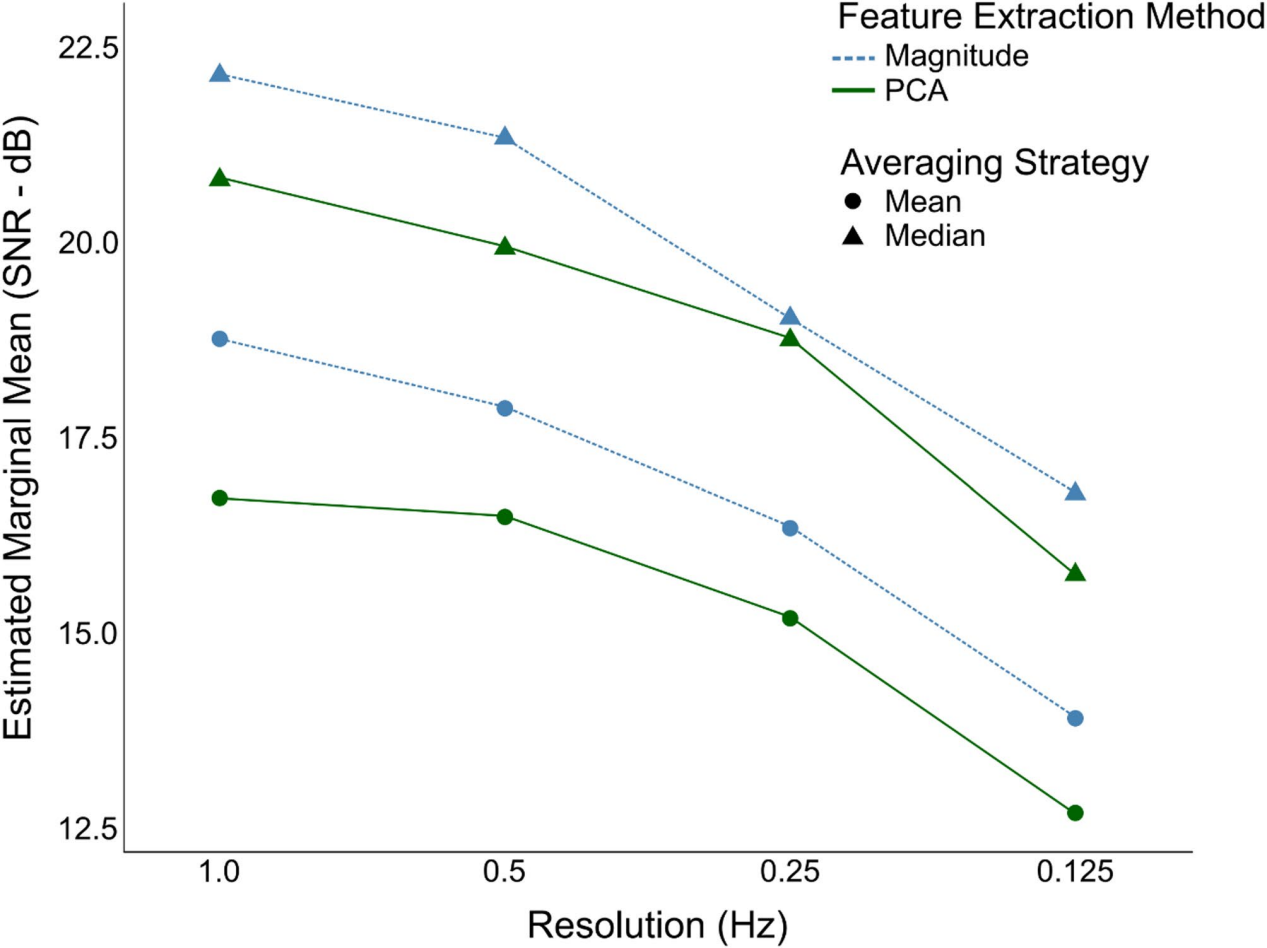
Estimated marginal means of log-transformed signal-to-noise ratios (SNRs, in decibels) across different resolutions (1 Hz, 0.5 Hz, 0.25 Hz, 0.125 Hz), plotted separately for Magnitude (blue) and PCA (green), and for averaging strategies Mean (dots) and Median (triangles).

Figure 3 shows that SNR was strongly affected by the Averaging strategy, with Median-based methods yielding markedly higher values than Mean-based ones, *F*(1,31) = 202.04, *p* < 0.001, η^2^_G_ = 0.112. Although Magnitude-based methods produced slightly higher SNRs than PCA, this difference did not reach significance: *F*(1,31) = 3.13, *p* = 0.087, η^2^_G_ = 0.016. The main effect of Resolution was also significant, *F*(1.97, ∼61.2) = 66.39, *p* < 0.001, η^2^_G_ = 0.122. However, none of the interaction terms were significant, including the Resolution × Averaging strategy interaction (*F*(3,93) = 1.45, *p* = 0.23), suggesting that the advantage of the Median-based method was consistent across resolutions. Post-hoc comparisons for the Median-based method revealed that 0.125 Hz yielded significantly higher SNRs than 1 Hz, 0.5 Hz, and 0.25 Hz (all *p* < 0.001). Additionally, 0.25 Hz was significantly higher than both 1 Hz and 0.5 Hz (both *p* < 0.001), while 0.5 Hz was modestly higher than 1 Hz (*p* = 0.0185). Overall, the combination of Magnitude and Median at 1 Hz–0.5 Hz yielded the strongest frequency-specific responses with minimal noise.

**Fig. 4:**
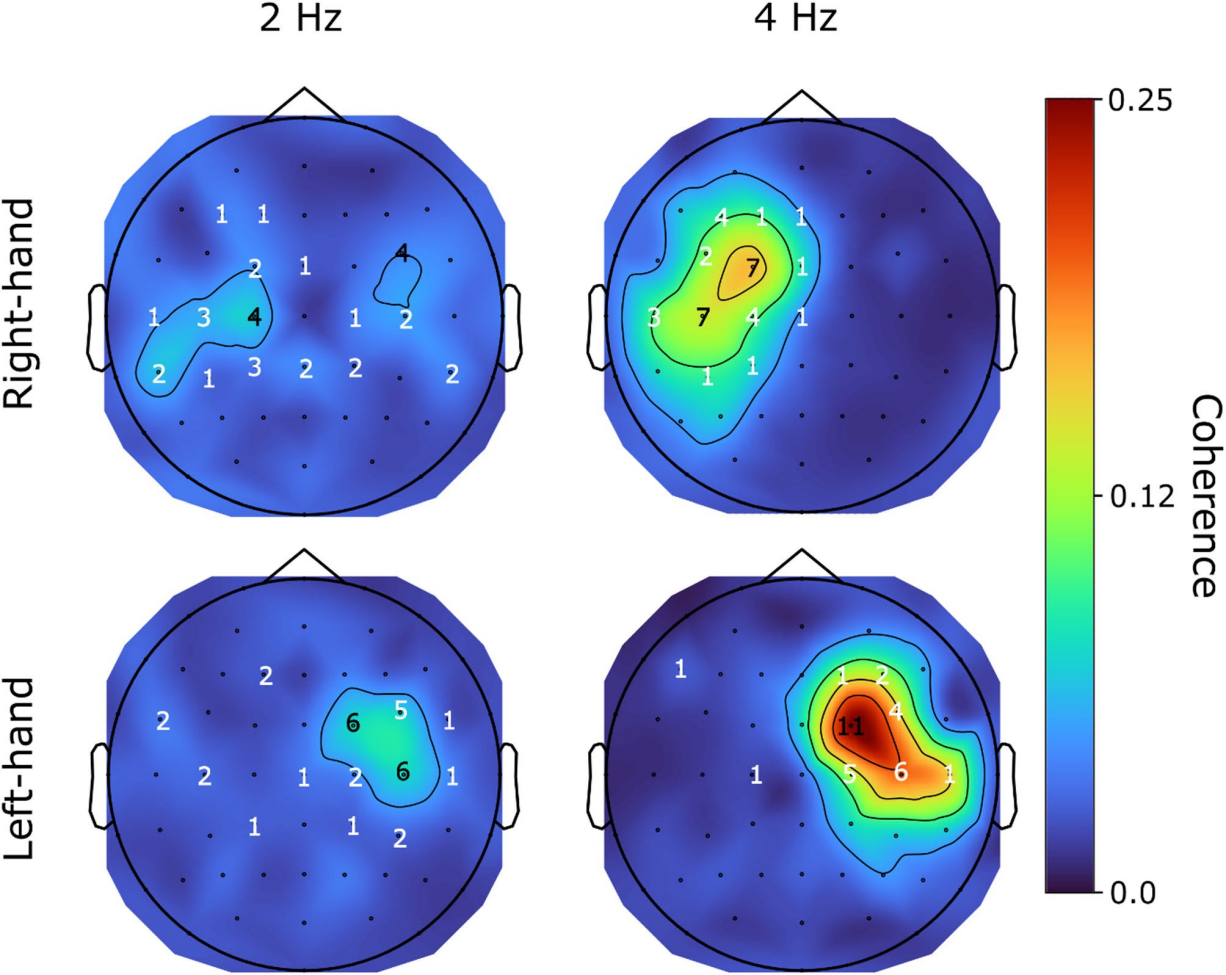
Topographic distributions of the group mean (*n* = 32) CKC peaks at 2 Hz (left column) and 4 Hz (right column) for right-hand movement (top row), and left-hand movement conditions (bottom row).

Based on the preceding comparison of coherence metrics, the Magnitude-Median method at 0.5 Hz resolution was selected as the optimal configuration for all subsequent analyses. Using this configuration, the topographic distribution of the group mean CKC at 2 Hz and 4 Hz for the right- and left-hand movements (Fig. 4) corresponds well to those reported in the literature in studies using finger-^21^ or hand-movement-based^20^ CKC derivations. CKC peaked at electrode sites above the hand area of the SM1 cortex contralateral to the moved hand at 2 Hz and 4 Hz. However, CKC at 2 Hz in the right-hand condition exhibited less consistent results, with peaks above the C1, C3 electrodes and a peak at the right hemisphere between FC4 and C4 electrodes.

**Fig. 5:**
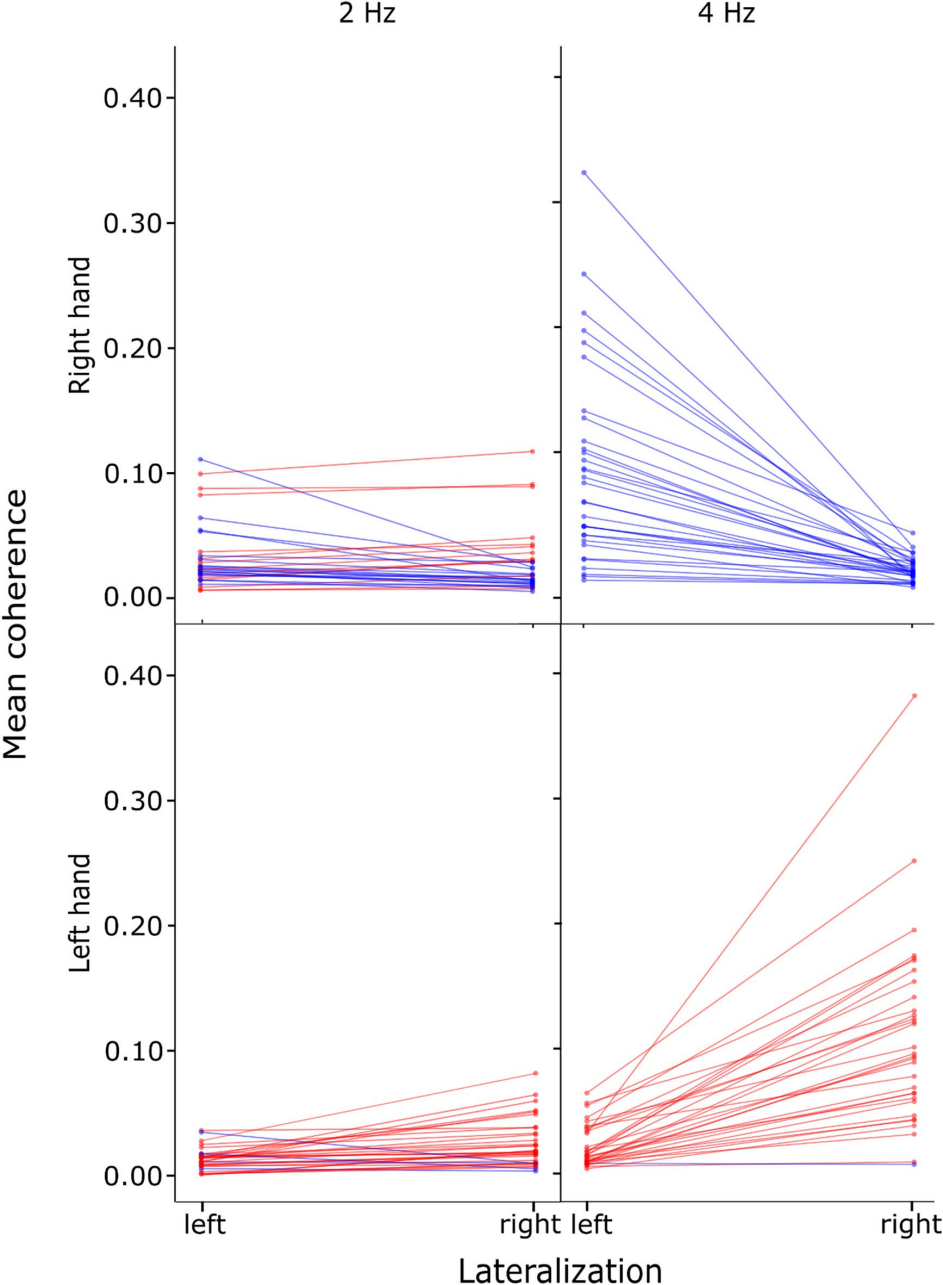
2 Hz (left) and 4 Hz (right) CKC lateralization for right-hand (top row) and left-hand (bottom row) movement conditions. Individual participant (*n* = 32) mean CKC over the left and right electrode sets (see Fig. 1) is shown for each participant: blue lines correspond to left-lateralized, red lines right-lateralized mean CKC for the given participant.

Between-hemisphere CKC comparisons showed clear lateralization (see Fig. 5). Significant differences corresponding to increased mean CKC on electrodes on the side contralateral to the moving hand (in comparison to the ipsilateral electrodes) were found in all comparisons except for the 2 Hz CKC for the right-hand movement (see Table 1).

**Table 1:** Statistical comparison of CKC lateralization between contralateral and ipsilateral electrodes for right- and left-hand movement conditions at 2 hz and 4 hz.

Condition	Shapiro-Wilk normality test (W)	*p*-value	Wilcoxon signed rank test (V)	*p*-value
Right hand 2 Hz	0.796	< 0.001***	311	0.39
Right hand 4 Hz	0.856	< 0.001***	528	< 0.001***
Left hand 2 Hz	0.9	< 0.001***	476	< 0.001***
Left hand 4 Hz	0.839	< 0.001***	527	< 0.001***

Note. *p < 0.05, **p < 0.01, ***p < 0.001.

To evaluate the frequency specificity, coherence values at target frequencies (2 Hz and 4 Hz) were compared with the neighboring frequencies of 1.5 Hz, 2.5 Hz for 2 Hz and 3.5 Hz, 4.5 Hz for 4 Hz. The Shapiro-Wilk test revealed non-normality for the 2 Hz vs. 3 Hz comparison (*p* = 0.004), while the 4 Hz vs. 3 Hz comparison was normally distributed (*p* = 0.238).

**Fig. 6:**
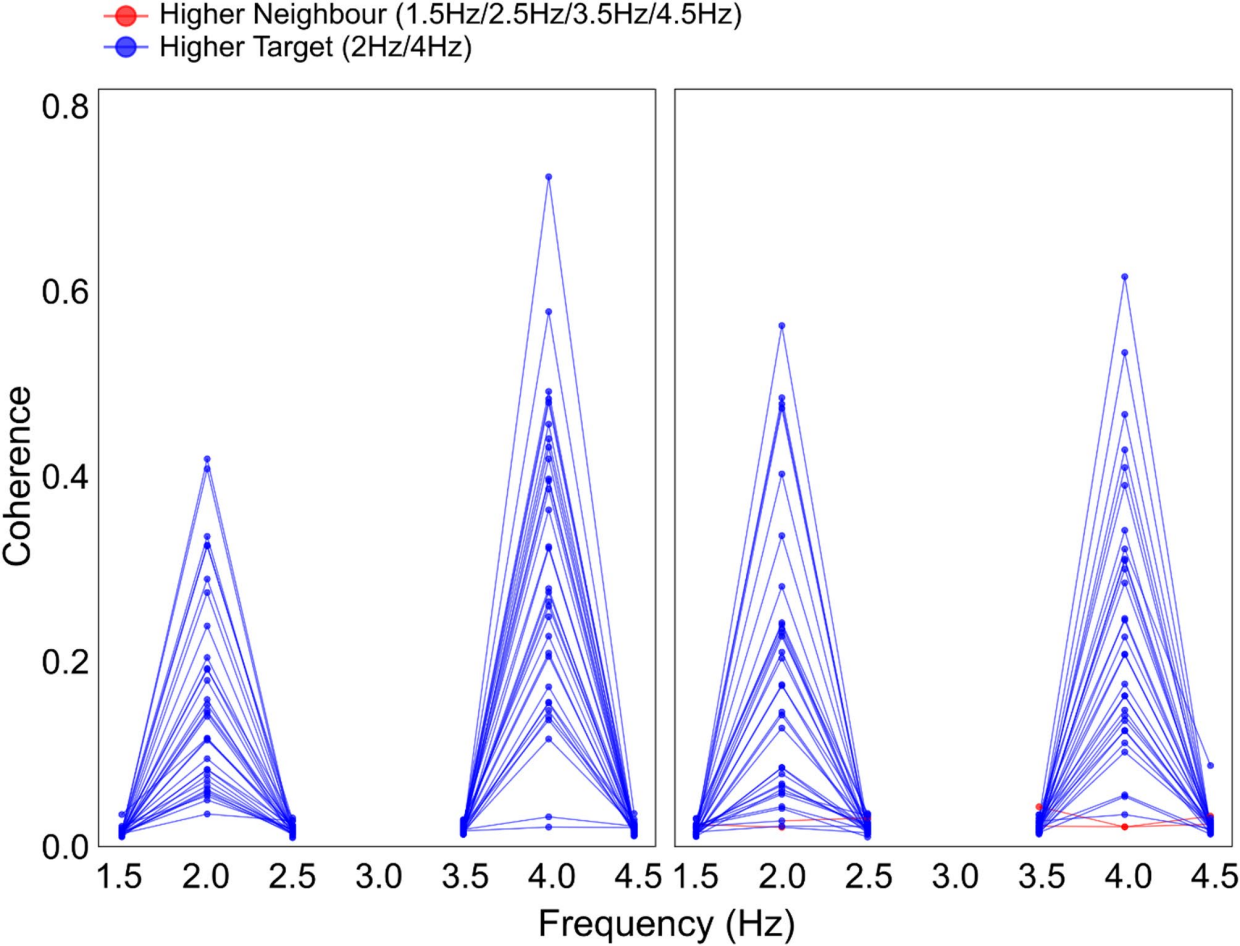
Individual coherence comparisons across frequencies for each hand condition. Coherence at target frequencies (2 Hz and 4 Hz) was compared with coherence at the neighboring 1.5, 2.5, 3.5 and 4.5 Hz frequencies for right- and left-hand conditions using paired statistical tests. Colored lines and points represent whether coherence was higher at the target (blue) or neighboring frequency (red).

Accordingly, a Wilcoxon signed-rank test was used for the 2 Hz condition, and a paired t-test for the 4 Hz condition. Both tests revealed significantly higher coherence at the target frequency compared to neighboring frequencies (see Table 2; Fig. 6), supporting the presence of frequency-specific CKC responses.

**Table 2:** Frequency specificity of CKC responses at 2 hz and 4 hz compared to neighboring frequencies.

Condition	Shapiro-Wilk normality test (W)	*p*-value	Test used	Statistic	*p*-value
2 Hz vs. 1.5 Hz	0.891	0.004 **	Wilcoxon signed-rank	V = 528	< 0.001 ***
2 Hz vs. 2.5 Hz	0.906	0.009 **	Wilcoxon signed-rank	V = 528	< 0.001 ***
4 Hz vs. 3.5 Hz	0.964	0.359	Paired t-test	t = 11.29	< 0.001***
4 Hz vs. 4.5 Hz	0.962	0.307	Paired t-test	t = 11.68	< 0.001***

Note. *p < 0.05, **p < 0.01, ***p < 0.001.

Spearman’s rank correlations calculated to examine relationships between maximum CKC values in the right-hand and left-hand conditions at 2 Hz and at 4 Hz, as well as between 2 Hz and 4 Hz in right-hand conditions and between 2 Hz and 4 Hz in left-hand conditions showed one significant relationship only: the correlation between the left-hand and right-hand 2 Hz condition CKCs (see Table 3).

Separate Spearman’s rank correlation analyses conducted to examine the relationship between maximum CKC values in dominant-hand and non-dominant-hand conditions showed one significant correlations: between dominant and non-dominant hand conditions at 2 Hz CKCs (see Table 4). Correlations between handedness absolute value score and maximum CKC values measured for the dominant and non-dominant hands at 2 Hz and 4 Hz revealed one significant correlation: a positive correlation between handedness score and CKC at 2 Hz for the dominant hand (see Table 5).

**Table 3:** Spearman correlations between right-hand and left-hand conditions at 2 hz and 4 hz. Only correlation coefficients (ρ) are reported; statistical significance is indicated by asterisks, with *p*-values uncorrected for multiple comparisons.

Condition	Right hand 2 Hz	Right hand 4 Hz	Left hand 2 Hz
Right hand 4 Hz	0.237	-	-
Left hand 2 Hz	0.48**	-	-
Left hand 4 Hz	-	0.219	0.038

Note. *p < 0.05, **p < 0.01, ***p < 0.001.

**Table 4:** Spearman correlations between dominant-hand and non-dominant-hand conditions at 2 hz and 4 hz. Only correlation coefficients (ρ) are reported; statistical significance is indicated by asterisks, with *p*-values uncorrected for multiple comparisons.

Condition	Dominant hand 2 Hz	Dominant hand 4 Hz	Non-dominant hand 2 Hz
Dominant hand 4 Hz	0.341	-	-
Non-dominant hand 2 Hz	0.476**	-	-
Non-dominant hand 4 Hz	-	0.210	− 0.056

Note. *p < 0.05, **p < 0.01, ***p < 0.001.

**Table 5:** Spearman correlations between handedness absolute value score and CKC measured for the dominant and non-dominant hands at 2 hz and 4 hz, note that p-values are uncorrected for multiple comparisons.

Condition	Handedness score	
	Rho	p
Dominant hand 2 Hz	0.398	0.024*
Dominant hand 4 Hz	0.28	0.121
Non-dominant hand 2 Hz	0.088	0.630
Non-dominant hand 4 Hz	−0.152	0.407

Note. *p < 0.05, **p < 0.01, ***p < 0.001.

## Discussion

The goal of the present study was to introduce a physiotherapist-assisted wrist-movement-based protocol suitable for stroke patients and demonstrate its utility for the measurement of CKC using EEG in healthy adults.

Movement analyses showed that the “visual metronome” allowed the physiotherapist to closely control and maintain the 2 Hz target hand-movement frequency, typically compensating for deviations in the next movement cycle. Deviations were relatively small, with 50% of the periods within a 25 ms range around the prescribed 500 ms. It must be noted, however, that right hand movement was systematically less regular than that of the left-hand, manifested as a slightly (0.6 ms) shorter typical movement period and larger variability (6 ms longer inter-quartile range). This suggests that the inherent asymmetry of the stimulation arrangement setup due to the hand-preference of the physiotherapist is difficult to avoid, and differences in the movement itself may affect CKC measurement as well. While the movement data and coherence peaks suggest that physiotherapist-guided stimulation - aided by the visual metronome - was sufficiently precise for CKC measurement, temporal accuracy is inherently lower than that of mechanical actuators. This limitation may be particularly relevant in light of findings that CKC strength is enhanced by temporally regular stimulation compared to irregular input, as demonstrated by Piitulainen et al.^37^. Future studies should directly compare manually and mechanically delivered stimulation to characterize trade-offs between temporal accuracy and clinical adaptability, helping to further validate the feasibility of manual stimulation for CKC assessment.

The movement and coherence analyses highlight the precision and stability of the physiotherapist-assisted passive wrist movement. The clear CKC peaks at the 2 Hz movement frequency and its first harmonic (4 Hz) observed in the coherence spectra across all methods and resolutions suggest that the close control of the movement supported by the “visual metronome” movement cycle was well-maintained. These findings confirm the temporal accuracy of the movement and the effectiveness of the physiotherapist-assisted wrist-movement-based protocol. The median-based method considerably improved CKC signal-to-noise ratio – improving separation between target and neighboring frequencies. The highest signal-to-noise ratios were observed at the coarser 1 Hz and 0.5 Hz frequency resolutions. Although 0.25 Hz and 0.125 Hz resolutions yielded somewhat lower SNRs, coherence peaks at target frequencies were clearly distinct from coherence signals at neighboring frequencies (see Fig. 2). This suggests that even though SNR may be reduced, the finer frequency resolutions may allow the separation of multiple, close-by movement frequencies in future studies. These findings highlight the importance of optimizing EEG-based methods, particularly enhancing spatial resolution through techniques like the surface Laplacian and regularization. Overall, these results contribute to improving EEG-based CKC methods, enhancing their accessibility and reliability for future research.

CKC patterns were similar to those reported by Piitulainen et al.^21^ for finger- and Smeds et al.^20^ for hand-movements, with peaks emerging at 2 and 4 Hz, while coherence at neighboring frequencies remained consistently lower across all conditions (see Table 2; Fig. 6), suggesting that the CKC response is robustly tuned to the specific movement frequencies. This pattern supports the hypothesis that CKC responses are finely tuned to the periodicity of actual movement input, rather than arising from more general or broadband sensorimotor activity. Moreover, CKC was strongest at electrode sites contralateral to the moved hand, above the somatosensory cortex corresponding to the hand’s cortical representation. In agreement with the results reported by Smeds et al.^20^ the CKC topographical peak distribution at 2 Hz was less consistent than at 4 Hz (see Fig. 4), with no clear evidence for lateralization in the 2 Hz right-hand movement condition. Smeds and colleagues^20^ speculated that the more marked peak at the first harmonic (4 Hz) reflected the sensitivity of the somatosensory cortex to the absolute (i.e., unsigned) movement velocity, which is reached twice (with opposite signs) within a single movement period, suggesting that wrist flexors and extensors contract sequentially during each movement cycle. This cyclic change in muscle activation might make neural activity associated with controlled acceleration by either muscle group indistinguishable at the scalp level (hence a higher 4 Hz CKC). However, this raises the question of the origin of the 2 Hz peaks. Repetitive joint movements consist of alternating stretches of flexor and extensor muscles and thus generate at least two bursts of proprioceptive input per cycle, which likely explains the 4 Hz component. The 2 Hz CKC peak may reflect input from a single muscle group (e.g., the flexors), or represent cortical responses that differentiate between directional or temporally specific aspects of the movement cycle. In addition to muscle spindles, joint receptors such as Pacinian corpuscles - being sensitive to joint acceleration and rapid compressive forces^58,59^ - may also contribute to the neural encoding of cyclic mechanical input and account for part of the 2 Hz signal. It is also important to reconsider the common assumption that finger movements provide inherently superior CKC signals due to their relatively large somatosensory cortical representation^23,60^. Although this anatomical characteristic likely contributes to CKC signal strength, wrist movements may activate a broader set of proprioceptors. Because many of the muscles that control finger movement are located in the forearm and pass through the wrist, passive wrist movement results in sequential stretching not only of the wrist muscles but also of the finger flexors and extensors. This broader engagement may yield more widespread or stronger afferent input compared to isolated finger movement, engaging both wrist- and finger-related proprioceptors, similar to the summation effects reported by Hakonen et al.^61^ for multi-finger stimulation. However, the overall CKC magnitude observed with EEG remains lower than typically reported in MEG studies. The reason for this discrepancy is not yet fully understood but may stem from a combination of methodological and anatomical factors. One speculation relates to the differing sensitivities of MEG and EEG to cortical source orientation. While MEG is most sensitive to tangential sources^19^ - such as those in area 3b within the central sulcus, EEG captures both tangential and radial activity, including from area 1 on the postcentral gyrus. Beside the possibility that proprioceptive input could be differentially processed or represented in these regions, the superposition of the differentially oriented electric fields may lead to partial cancellation, thus contribute to the relatively reduced CKC amplitudes in EEG-based studies^62,63^. Additionally, the variability in movement during the right-hand condition might have contributed to the inconsistent lateralization observed at 2 Hz.

While the study was not primarily designed to assess individual differences, we explored whether CKC strength was consistent across hands or associated with handedness. Significant correlations were observed between CKC values at 2 Hz for the left and right hands (ρ = 0.48), and between dominant and non-dominant hand 2 Hz responses (ρ = 0.476), suggesting a degree of within-subject consistency. This could reflect stable individual traits in proprioceptive processing or cortical responsivity, aligning with previous reports of CKC stability across sessions and hands in healthy adults^32^as well as longitudinal consistency in clinical populations^25^. Additionally, CKC at 2 Hz in the dominant hand was weakly associated with handedness score (ρ = 0.398, *p* = 0.024). Notably, all these associations were observed at 2 Hz - the movement frequency used in the task - and not at 4 Hz, possibly reflecting stronger tuning of CKC responses at the actual stimulation frequency. Moreover, the sample was primarily right-handed, which may have contributed to the asymmetry in results across dominant and non-dominant hand conditions. Although none of these correlations would survive correction for multiple comparisons and should be interpreted with caution, they may hint at stable, trait-like characteristics of CKC. This possible consistency is particularly interesting in light of known asymmetries in the structure and function of the sensorimotor cortices related to hand dominance, such as differences in the shape of the central sulcus and lateralization of proprioceptive processing, which may contribute to interindividual variation in cortical responses^64–66^. Future research with larger samples and hypothesis-driven designs is needed to clarify the reliability and functional relevance of these associations. The present study demonstrated the feasibility of characterizing proprioception in a stimulation setup that can accommodate individual variability in movement ability to suit patients participating in post-stroke rehabilitation. This protocol offers several appealing attributes that position it as a promising marker for proprioceptive function. By involving a physiotherapist, the protocol enables personalized movement and immediate adaptations, while the combination of passive wrist movement and EEG-based measurements with a short recording duration ensures a more practical approach suitable for participants exhibiting diverse motor abilities and physiological conditions. Meanwhile, the visual aid allowed the maintenance of regular stimulation necessary for the measurement of CKC. Asymmetries in the stimulation, nonetheless, introduced slight between-hands differences in movement regularity, which should be kept in mind when interpreting potential between-hands differences.

## Supplementary Information

Below is the link to the electronic supplementary material.

Supplementary Material 1

Supplementary Material 2

## Data Availability

The dataset generated and analyzed in the current study will be made available upon publication at https://figshare.com/s/95c525aaac61c58b2d7b.
