# Supplementary material for: Physiotherapist assisted wrist movement protocol for EEG-based corticokinematic coherence assessment
Source: Sci Rep. 2025 Sep 26;15:33104. doi: 10.1038/s41598-025-17330-5 (PMC12475183; doi:10.1038/s41598-025-17330-5)
Supplement: Supplementary file 1 — Supplementary Material 1 [file 41598_2025_17330_MOESM1_ESM.docx]

*Supplementary Video S1. Overview of the Experimental Setup and Physiotherapist-Guided Movement Procedure*

This short video demonstrates the physiotherapist-assisted wrist movement protocol used in the experiment. The clip provides an overhead view of the setup, showing a physiotherapist passively moving the participant's wrist in sync with a visual metronome displayed on a tablet positioned beneath the participant’s hand. This setup was designed to produce rhythmic, 2 Hz wrist movements during the EEG recordings.

Please note that, for illustrative purposes, several components used during actual data collection are not visible in this demonstration. Specifically, EEG caps, electrode cables, the hand-acceleration sensors (used to compute CKC), the elastic straps securing the participant's arm, and the curtain used to block the participant’s view of the movement have been omitted to avoid obstructing the view of the hand and the movement. Despite these omissions, the video accurately represents the procedure, hand positioning, and physiotherapist-participant interaction central to the stimulation protocol.
